# Palliative Free Flap Surgery for Plantar Sarcoma: A Case Report and Literature Review

**DOI:** 10.7759/cureus.30488

**Published:** 2022-10-19

**Authors:** Kosuke Masaoka, Satoka Tokuhara, Kota Tsuchiya, Yuki Komatsu, Shunsuke Sakakibara, Tadashi Nomura, Hiroto Terashi

**Affiliations:** 1 Department of Plastic Surgery, Kobe University Graduate School of Medicine, Kobe, JPN; 2 Department of Plastic Surgery, Japanese Red Cross Kobe Hospital, Kobe, JPN; 3 Department of Plastic Surgery, Shinko Hospital, Kobe, JPN

**Keywords:** dynamic skin tissue perfusion pressure test, quality of life, palliative care, palliative surgery, free flaps

## Abstract

We report a case of palliative surgery in a 73-year-old patient with metastatic plantar sarcoma. The patient underwent resection and irradiation of an undifferentiated spindle cell sarcoma in the right plantar region. The wound was not closed and systemic metastases were observed. The chief complaint of the patient on his first visit to our department was difficulty walking due to pain in the right plantar region. Since we were unsuccessful in relieving the pain with conservative treatment, we decided to perform a palliative free tissue transfer to the right plantar. The surgery was successful, the skin ulcer healed, and the pain was relieved after the surgery. When performing palliative surgery, more detailed preoperative management and planning are necessary to achieve a successful outcome. The selection of the flaps according to the local lesion and metastatic lesions and changes in the local hemodynamics should be considered when planning.

## Introduction

In patients with advanced malignancies, such as unresectable or metastatic cancer, treatment of local lesions must be emphasized to reduce pain and bleeding, which leads to spiritual and psychological well-being. Palliative surgery is performed when there is a prospect that the operation may improve the quality of life of patients suffering from difficulties [1]. In this report, we present a case of palliative surgery involving free-flap reconstruction of the right plantar after resection and radiation therapy (RT) for plantar sarcoma.

## Case presentation

History

The patient was a 73-year-old female who first visited the orthopedic department of our hospital complaining of a right plantar mass. Percutaneous biopsy revealed sarcoma, and extended resection was performed. The pathological diagnosis of the resected tumor was an undifferentiated spindle cell sarcoma. The wound was not closed primarily, and conservative management was continued. In addition, 70 Gy of local radiation was administered because residual tumor cells were confirmed in the resected margin.

A year after the surgery, metastases to the left gluteal muscle, spine, soft tissue of the right thigh, body and tail of the pancreas, right inguinal lymph node, and soft tissue of the right arm were observed. All metastatic lesions were resected except for the lesion in the right thigh. Chemoradiation therapy (CRT) was performed on the spinal lesion, and radiation therapy (RT) was performed on the right thigh after resection. The patient also had advanced metastatic lesions in the stomach. Instead of extended resection, local resection of the stomach was performed to prevent postoperative complications. Five years after the primary surgery to resect the right plantar lesion, the open wound had not healed, and the patient was referred to our department for a complaint of difficulty in walking due to increasing pain in the right plantar region.

Examination and imaging

**Figure 1:**
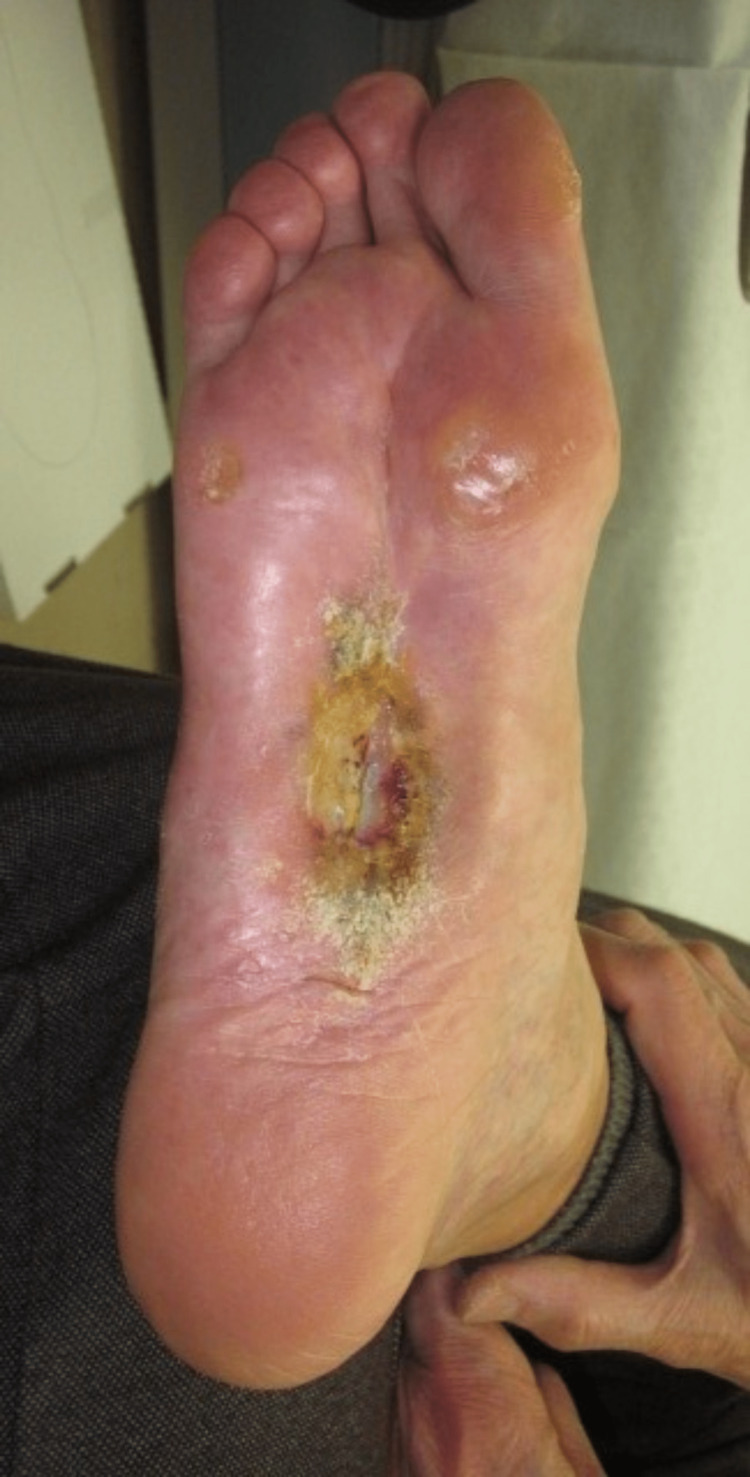
Initial examination A stage 4 based on CTCAE v5.0 Grade Severity Score skin ulcer in the right plantar was observed. The size was 8 cm × 4 cm and the granulation inside was poor.

**Figure 2:**
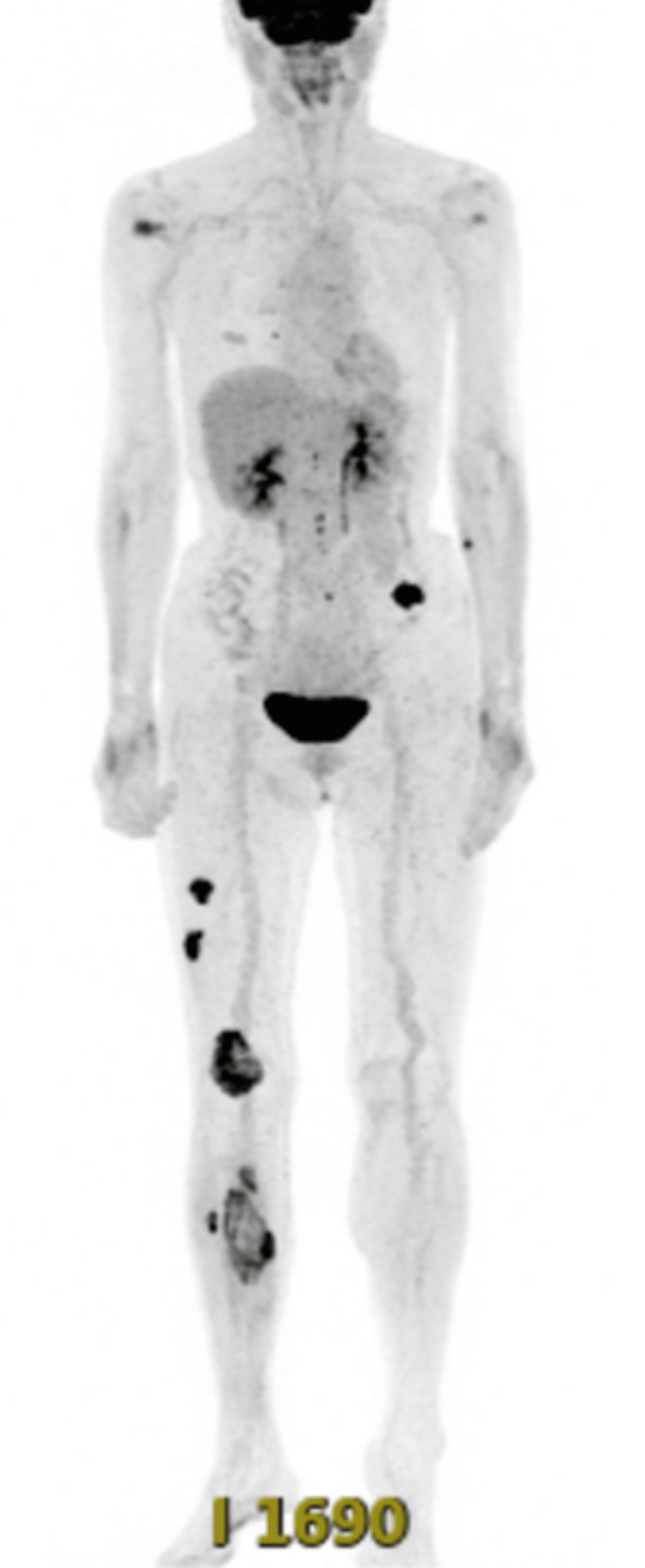
PET-CT PET-CT revealed metastases to the lungs, right lower limb, left gluteal region, mediastinal and supraclavicular lymph nodes.

A stage 4 based on CTCAE v5.0 Grade Severity Score, measuring 8 cm × 4 cm of the right plantar region, was observed at the time of initial examination. Granulation inside was poor (Figure 1). Positron emission tomography (PET-CT) revealed extensive distant metastases (Figure 2).

Pre-operative planning

We treated the patient with conservative management using an orthosis and analgesics, which was not effective in relieving pain when walking. The score of the leg was 1/10 at rest and 10/10 at load. As relieving the pain was not successful, we decided to perform palliative surgery.

**Figure 3:**
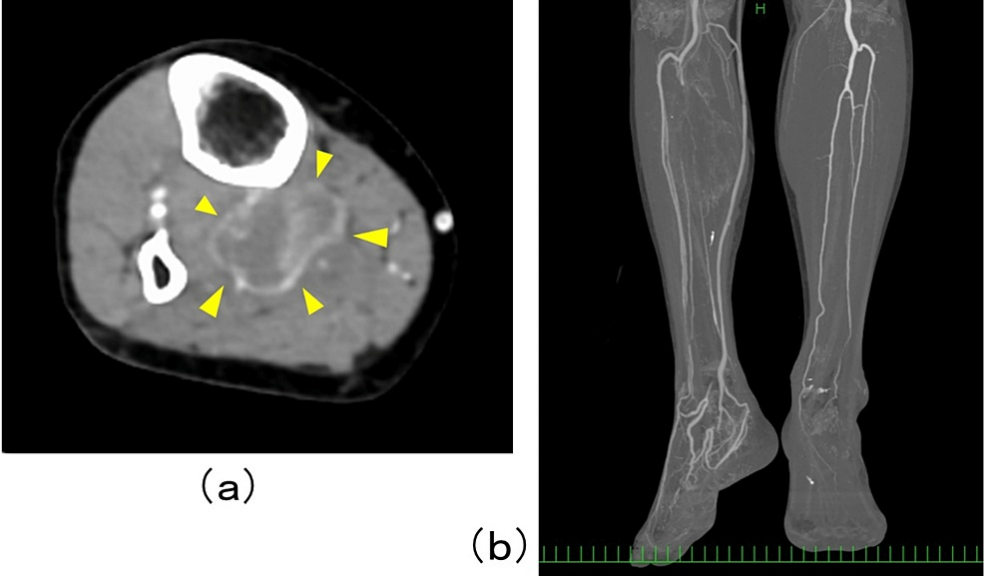
Contrast enhanced CT of the lower leg Preoperative contrast enhanced CT revealed a massive metastatic lesion (yellow arrow) in the proximal area of the right lower leg (a), occluding the tibial fibular trunk of popliteal artery and early staining of the great saphenous vein (b).

We elected to perform free tissue transfer to reconstruct the plantar, considering the blood supply to the affected leg. Healing of the plantar wound by skin grafting or a local flap was not feasible because the ulcer of the right plantar was not well granulated and the blood supply to the right leg was supplied only by the anterior tibial artery. Preoperative contrast-enhanced computed tomography (CT) revealed a massive metastatic lesion in the proximal area of the right lower leg, occluding the tibial fibular trunk of the popliteal artery and early staining of the great saphenous vein (Figure 3).

**Figure 4:**
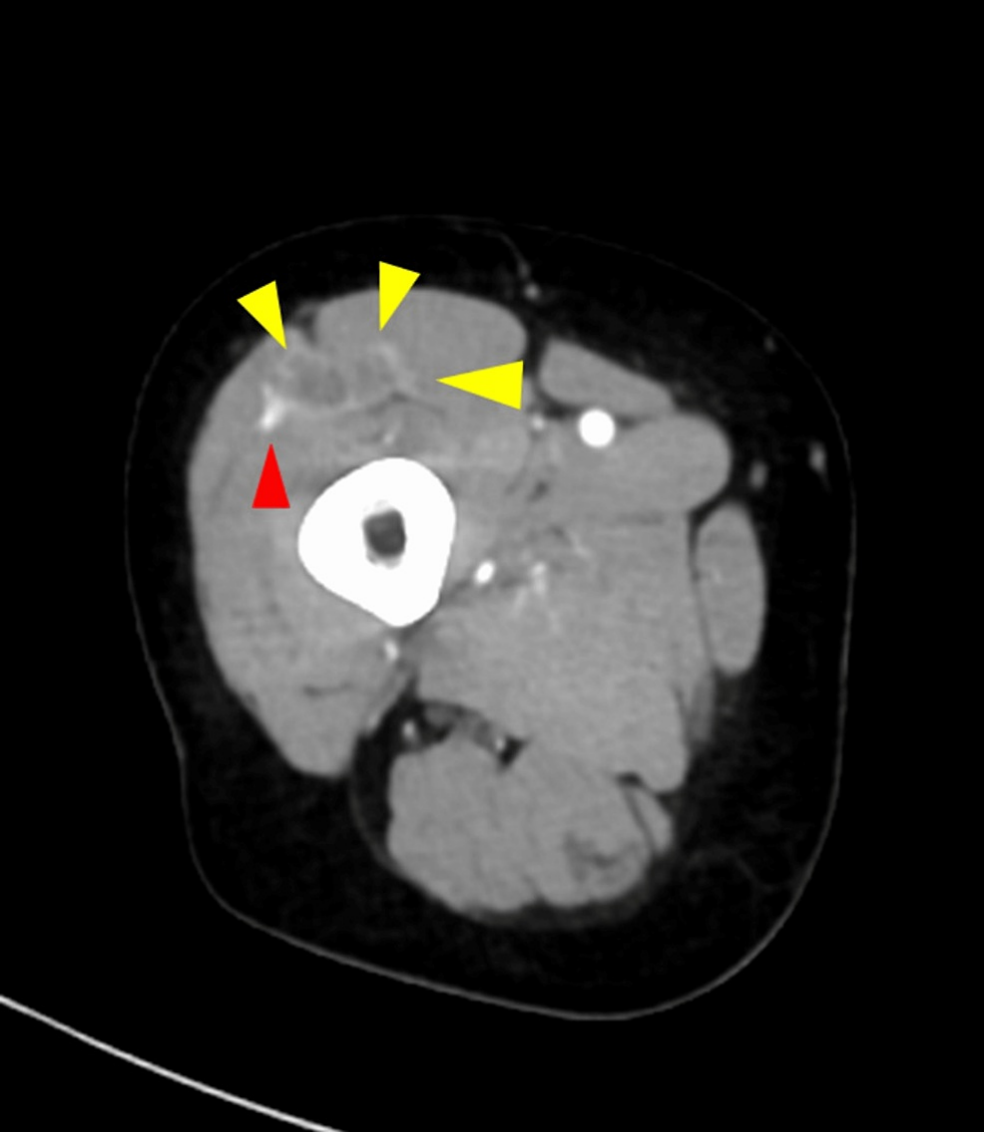
Contrast-enhanced CT of the thigh Metastases (yellow arrow) around the right lateral femoral circumflex artery (red arrow) were observed.

We selected an anterior lateral thigh flap from the contralateral side of the affected leg. Metastases were observed around the right lateral femoral circumflex artery (Figure 4). The left latissimus dorsi flap was not appropriate for the operation because the muscle was affected by metastases and was extensively removed.

We selected the internal medial tarsal artery for pedicle anastomosis. While selecting the artery, we evaluated the skin perfusion pressure (SPP) of the anterior foot. Dynamic SPP was also measured when the responsible vessel, the medial tarsal artery, was compressed, and it was confirmed that the pressure did not decrease.

Operative course and outcome

**Figure 5:**
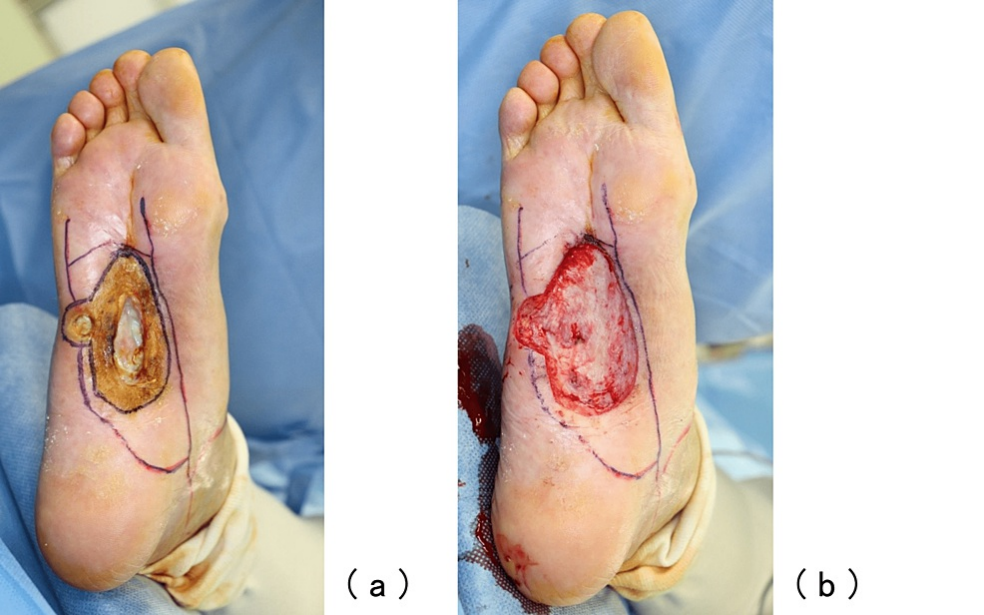
The first stage of operation The plantar artery was marked on the skin using ultrasonography before the operation, and considerable attention was paid not to injure the artery during the operation (a); at the end of the first stage of operation (b).

A two-stage surgery was performed. The first stage of the operation involved debridement and histopathological examination of the ulcer (Figure 5).

**Figure 6:**
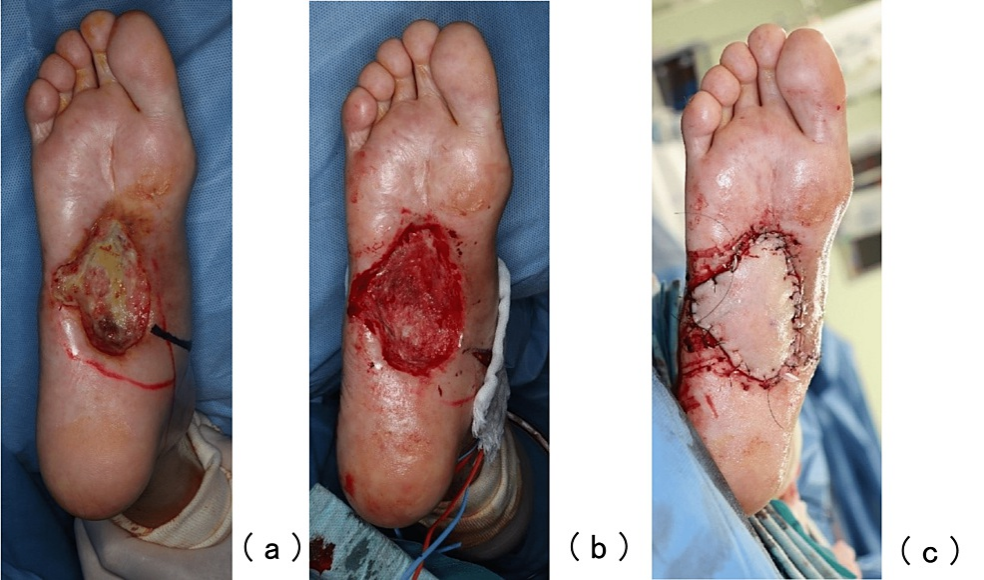
The second stage of operation Granulation was poor (a); debridement and a free tissue transfer of the left anterior lateral thigh flap was performed (b,c).

The plantar artery, which has blood supplied by the collateral artery, was marked on the skin using ultrasonography before the operation, and considerable attention was paid not to injure the artery during the operation. Negative pressure wound therapy (NPWT) was initiated on postoperative day 7 after confirming that there were no malignant findings in the resected ulcer. However, granulation was poor (Figure 6a). For the second operation, free tissue transfer of the left anterior lateral thigh flap was performed 27 days after the first stage of the operation (Figure 6b-6c), since granulation by NPWT was not enough to close the wound. The artery and vein pedicles of the flap were anastomosed end-to-end to the medial tarsal artery; the concomitant vein of the medial tarsal vein and a branch from the great saphenous vein were joined. Intraoperatively, the medial tarsal artery was found to flow in an anterograde fashion. The flap was successfully grafted without early postoperative complications. Although the metastases did not grow rapidly, systemic chemotherapy targeting slow-growing metastases was initiated after the second surgery.

**Figure 7:**
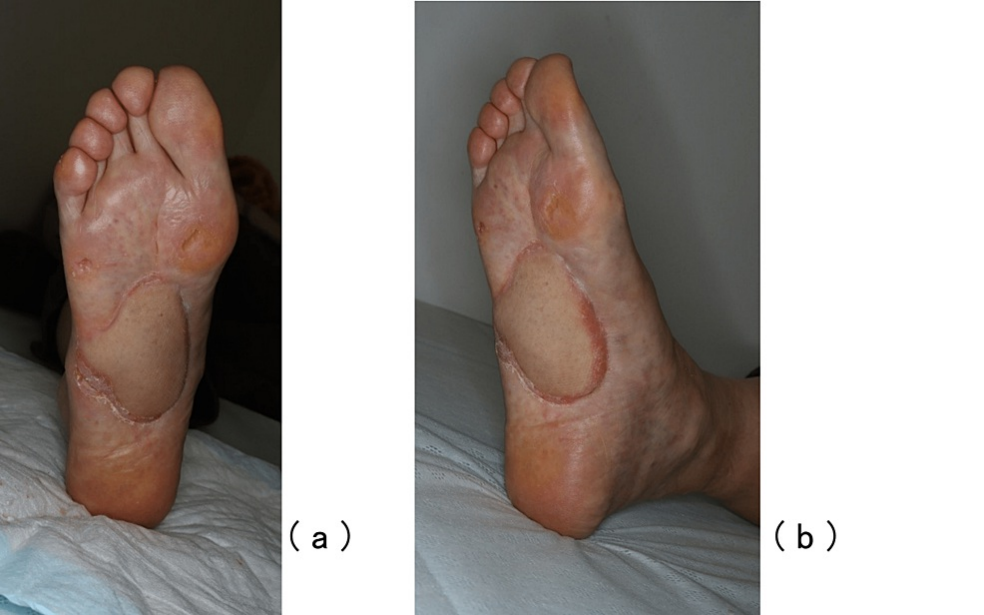
Ten months after the operation Frontal view of plantar (a) and oblique view (b). Recurrence of the ulcer was not observed 10 months after the operation.

In addition to chemotherapy, radiation was administered to the growing metastatic lesion in the right leg. No ulcer recurrence was observed 10 months after the operation (Figure 7). The pain from the ulcer was not completely relieved but decreased to NRS 1/10 at rest, 4/10 at loading, and 1-2/10 at loading with an orthosis. The patient is still alive and lives independently without any deterioration in ADL performance, although he requires a cane while walking.

Performance status improved from a preoperative score of 3 to a postoperative score of 1 on the PS scale.

## Discussion

Palliative care is generally considered to be non-curative and aims to improve the quality of life of patients and their families who face difficulties associated with chronic, incurable, life-threatening diseases [2]. One end of palliative care is the treatment of the burdensome symptoms of a chronic disease that may or may not be life-threatening, and the other end is end-of-life care. Palliative surgery is a part of palliative care, and it is performed when there is a prospect that the operation may improve the quality of life of patients suffering from difficulties such as pain [1]. Palliative surgery includes the reduction of offensive odor, pain, and bleeding [3-5] from the primary lesion of metastatic cancer. Cervical spine fixation for cervical spine metastases [6], surgery for pathological fractures [7], and gastrointestinal resection for malignant bowel obstruction [8,9] are all common palliative surgeries. In this case, surgery was performed not as end-of-life care but as a treatment for the burdensome symptoms of chronic disease.

In our case, no malignant findings were observed on the histopathological examination of the local lesion. This was suggestive of a radiation-induced ulcer that could not be cured by conservative management. The patient’s quality of life markedly decreased due to the pain, which made her unable to walk and required daily treatment of the wound. The operation we performed was considered palliative because it was intended to improve the patient’s quality of life and not alter the general disease course since the patient had systemic metastases.

Conditions that are suitable for palliative surgery were defined by Hanna et al. [1]. Since the patient's complaints are expected to be improved by surgery and the patient fully understands the disease and their condition. Our patient understood that the purpose of the operation was not intended to eradicate a malignancy. While any purposeful palliative operation should be mild and have low postoperative morbidity, we did not feel a less-involved procedure would be likely to achieve our operative goals and felt the free tissue transfer had a favorable risk-benefit analysis. When a malignant tumor is not eradicated, a long duration of surgical stress, which weakens innate immunity [10], may trigger malignancy progression. Therefore, we suggest that simple and easy surgical procedures should be selected whenever possible in performing palliative surgery.

There remains no clear consensus among surgeons regarding the indications for palliative surgery, especially in patients with malignancies. It has already been reported that free flaps are useful in palliative surgery [4,11]. It has been suggested that in cases involving radiation therapy, in which a local flap is difficult to perform, or in cases where a single palliative surgery is preferable for achieving a clinical goal, it may be effective to choose a free flap without considering a reconstruction ladder [11].

Negative pressure wound therapy was chosen between the exclusion of malignancy and the second surgery. Although negative pressure wound treatment can be rather painful and requires significant care, it is considered the best conservative treatment to promote granulation. However, it was determined that granulation was not sufficient and further treatment was needed.

For our patient, we chose a free flap as we were unable to close the chronic, non-healing wound of the debrided radiation ulcer. In addition, a local flap was not suitable for this patient considering the location of the lesion and blood supply, and we intended to epithelialize the wound within a short period of time.

The choice of technique was difficult because free flap transfer is a major procedure with considerable postoperative morbidity. In contrast, free dermal fat grafting and perifascial areolar tissue transfer are much more mild procedures that may promote angiogenesis and could have been performed as treatment. However, considering the possibility of inadequate angiogenesis and the time required for angiogenesis, these procedures were not performed in this case in order to shorten the treatment period.

It was difficult to determine whether to perform a free flap as a palliative surgery in this case. There was a possibility that the patient’s complaint of pain from the plantar ulcer would not disappear following the operation to close the wound, and there was a risk of postoperative complications of the transferred flap that could lead to unsuccessful outcomes.

In this case, even if the free flap was unsuccessful, it was not judged that the postoperative status of the ulcer would not be worse as compared to the preoperative condition. In addition, it was reasonable to close the wound using a free flap to improve the patient’s quality of life since the ulcer was the main cause of the decline in quality of life. Our operation was successful, although in a single case, we believe that our experience may be helpful in considering the indications for palliative surgery.

We believe that the indications and conditions for palliative surgery for non-healing wounds should always be determined on a case-by-case basis, depending on the local condition, general condition, and growth rate of the ulcer. We do not actively choose a free flap operation as it is preferable to perform simple and easy surgeries. Depending on the patient and the state of the tumor, a free flap should always be thought of as one of the options.

In this case, more detailed preoperative planning was required because the trunk of the posterior tibial fibular artery was occluded, and blood supply to the entire leg was through the anterior tibial artery alone. It was necessary to consider the blood flow to the anterior foot when performing debridement and selecting the anastomotic vessels. In addition, it was impossible to perform an ALT flap because of the metastases around the right lateral circumflex artery.

We had to consider these issues in preoperative planning, which were thought to be more complicated than those of a simple radiation ulcer.

In patients with systemic metastases, palliative surgery with flaps should be planned using imaging techniques such as PET-CT to evaluate metastasis to the location of the donor site of the flap and hemodynamics to the local lesion. In addition to ultrasonography, which is commonly used in the preoperative planning of a free flap operation, examination of deep lesions by contrast-enhanced CT, CT angiography, and MRI is essential in the preoperative planning of palliative surgery.

In this case, in addition to the usual anterior foot SPP test, we measured the dynamic SPP [12] under compression of the medial tarsal artery to confirm that the reduction in the blood flow of the artery would not affect the SPP of the forefoot. A simple and noninvasive dynamic SPP test was considered useful when selecting anastomotic vessels in a free flap operation with insufficient peripheral blood flow at the recipient site, as in this case.

## Conclusions

We report a case of palliative surgery using a free flap for a metastatic sarcoma with a radiation ulcer. In palliative surgery, careful preoperative planning is important, including flap selection and local hemodynamic changes. In this case, contrast-enhanced CT, PET-CT, and dynamic SPP tests were useful. We believe that our experience may be helpful in considering the indications for palliative surgery.
